# Towards a quantum therapy in cancer

**DOI:** 10.1002/ctm2.70411

**Published:** 2025-07-15

**Authors:** Frankie James Rawson

**Affiliations:** ^1^ Bioelectronics Laboratory Division of Regenerative Medicine and Cellular Therapies School of Pharmacy, Biodiscovery Institute University of Nottingham Nottingham UK

1

Bioelectricity^1^ and quantum^2^, ^3^ effects are increasingly recognised as fundamental mechanisms in biology underpinning various disease processes, offering new paradigms for therapeutic interventions. The article “Wireless electrical–molecular quantum signalling for cancer cell apoptosis”.^2^ introduces an innovative and potentially transformative approach to cancer treatment through the use of quantum biological tunnelling^4^ facilitated by wireless nano‐electrochemical tools. This groundbreaking research highlights the intersection of nanotechnology, quantum biology and oncology, providing a new paradigm for cancer cell apoptosis.

**FIGURE 1:**
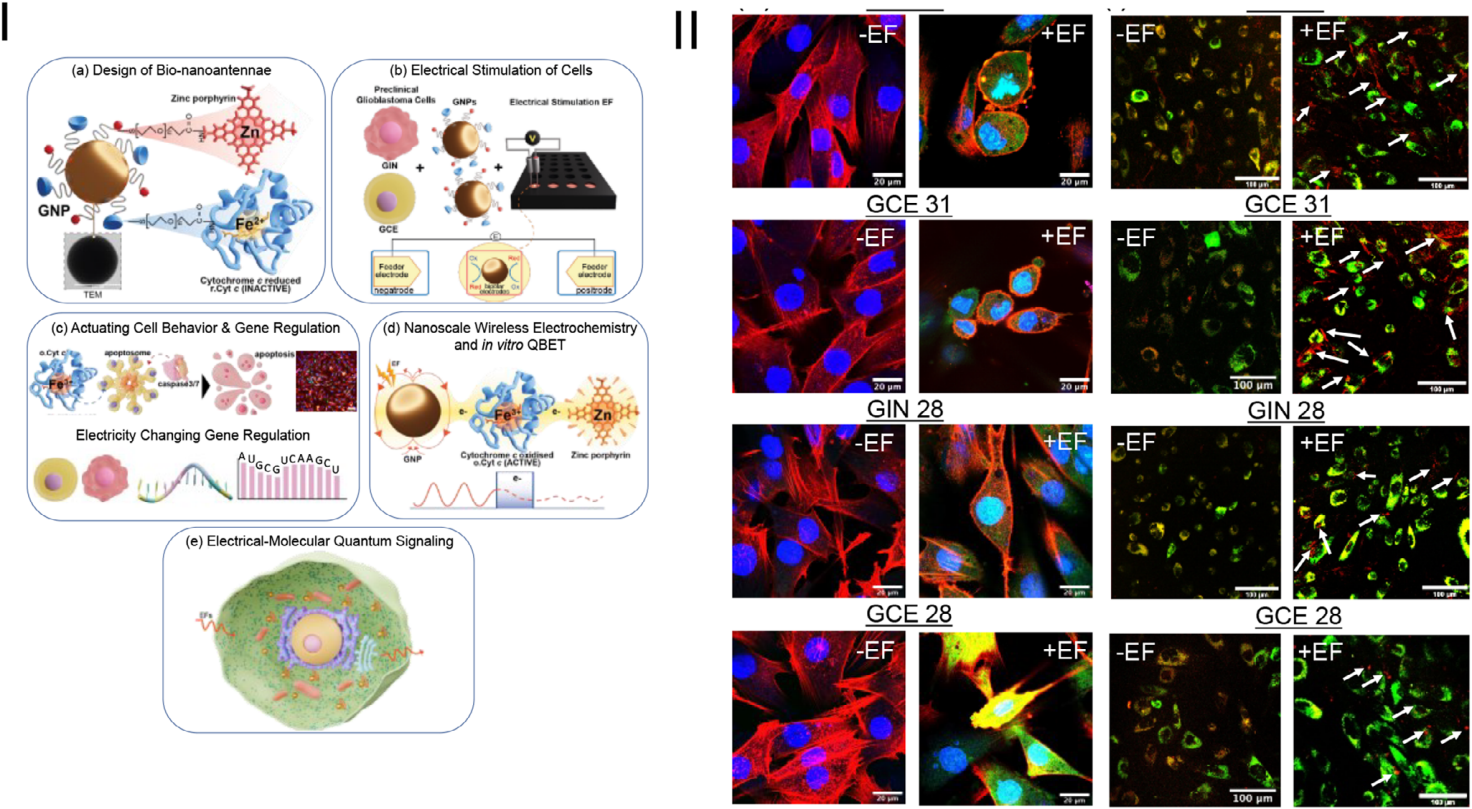
Quantum control of cancer cell apoptosis using bio‐nanoantennae and wireless electrochemical stimulation (I) Design of Bio‐nanoantennae: Bio‐nanoantennae (GNP100@r.Cyt c@Z) are synthesised by conjugating reduced cytochrome c (r.Cyt c) and zinc porphyrin (Z) to gold nanoparticles (GNP100). Primary patient‐derived glioblastoma cells (GIN and GCE) are incubated with these bio‐nanoantennae and subjected to a.c. electric fields (3 MHz, 0.65 V/cm) to facilitate nanoparticle uptake. This treatment induces intracellular wireless electrochemistry at the bio‐nanoantennae surface, triggering caspase‐3/7‐mediated apoptosis through gene regulation. Applying a.c. electric fields also switch the redox state of cytochrome c, demonstrating Quantum Biological Electron Transfer (QBET). The a.c.‐EF‐responsive bio‐nanoantennae facilitate electrical‐molecular quantum signalling in glioblastoma cells. (II) Confocal Microscopy Images: High‐magnification confocal microscopy images highlight caspase 3/7 activation in GIN and GCE cells post‐treatment, with cells stained for caspase 3/7 (green), actin (red), and nuclei (blue). Additionally, confocal images demonstrate the cytoplasmic localisation and endosomal escape of GNP100@r.Cyt c@Z in GIN and GCE cells following a.c. EF treatment, with cells stained using a late endosome dye (green) and white arrows indicating bio‐nanoantennae localisation. Adapted [1].

The study leverages gold bipolar nanoelectrodes functionalised with redox‐active cytochrome c and zinc porphyrin, termed bio‐nanoantennae, to regulate electron transport via a remote electrical input. This novel approach enabled selective triggering of apoptosis in patient‐derived cancer cells, specifically glioblastoma (GBM) cells, demonstrating a significant advancement in targeted cancer therapy (Figure 1).

The significance of this research lies in its ability to harness quantum mechanical effects for biological applications, specifically in modulating cellular functions. The development of bio‐nanoantennae capable of inducing apoptosis through wireless electrochemistry represents a novel tool in the arsenal against cancer. This method circumvents traditional limitations of drug delivery and specificity, offering a highly targeted approach that minimises collateral damage to surrounding healthy tissues.

**FIGURE 2:**
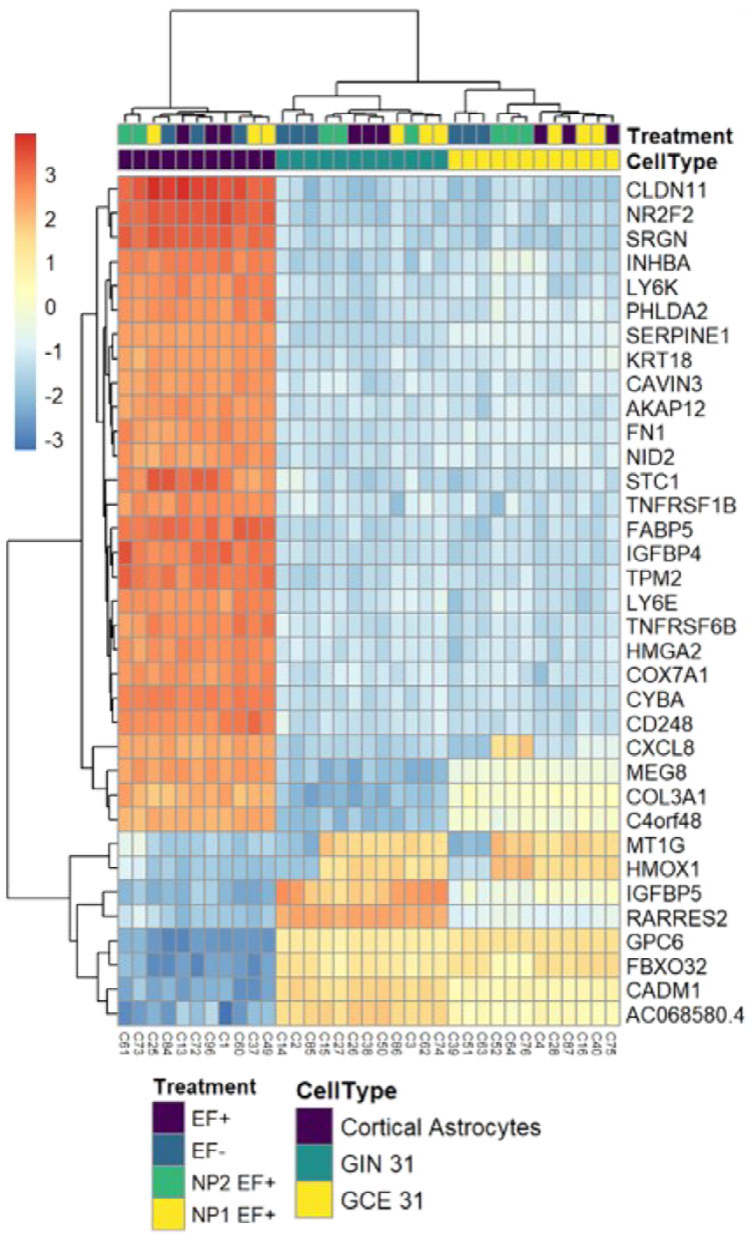
Gene expression changes post‐treatment: This heat map displays hierarchical clustering of the top 35 genes regulated after treatment with GNP100@r.Cyt c@Z for 8 h, followed by a.c. EF stimulation (3 MHz, 0.65 V/cm) for 2 h. Genes with the highest variance were selected after a variance‐stabilised transformation of the raw count matrix. Each row represents a gene, and each column represents a sample, with colours indicating differences from the row mean.

The research elucidated the underlying mechanism of action where the application of an alternating current (a.c.) electric field induces quantum biological tunnelling for electron transfer. This electron transfer is crucial for the redox state modulation of cytochrome c, switching from its reduced (Fe^2+^) to oxidised (Fe^3+^) state, which is known to trigger apoptotic pathways.^5^ The study's use of transcriptomics to analyse the gene expression changes further supports the specificity and effectiveness of the bio‐nanoantennae in inducing apoptosis (Figure 2).

An important finding of the study is the necessity of efficient endosomal escape for nanoparticles to exert therapeutic effects, aligning with current research from the Rawson group. They demonstrated that high‐frequency alternating current (HF‐AC) regulates endosomal escape, enhancing the bioavailability of therapeutic nanoparticles in the cytoplasm. Jain et al. support this,^2^, ^6^ showing HF‐AC facilitates gold nanoparticle (GNP) escape from endosomes in GBM cells, increasing their cytoplasmic concentration and efficacy.

The study presents a key technological advancement in designing gold bipolar nanoelectrodes, functioning as bio‐nanoantennae for wireless electrochemical interactions within cells. This successful implementation suggests potential for developing nanoscale devices for diagnostic and therapeutic purposes.

Clinically, this technology has vast potential. It offers a non‐invasive, wireless method to selectively induce apoptosis in cancer cells, potentially reducing the side effects of conventional chemotherapy and radiotherapy. This approach could be adapted for various cancer types, providing a versatile platform for personalised therapy.

Challenges remain for clinical translation: ensuring biocompatibility and long‐term safety of gold bipolar nanoelectrodes, scalable manufacturing for consistent quality, and controlling and delivering a.c. electric fields in clinical settings. Regulatory approval will require extensive clinical trials to prove efficacy and safety, which are time‐consuming and resource‐intensive.

Future research should focus on optimising bio‐nanoantennae for different cancers, exploring long‐term effects and safety in vivo, and integrating this technology with existing treatments to enhance efficacy and patient outcomes. Additionally, investigating its potential in other diseases where apoptosis regulation is beneficial is important.

This pioneering work merges quantum biology and nanotechnology, advancing our understanding of cellular electron transport and paving the way for novel, minimally invasive cancer treatments. The potential for clinical translation holds promise for significant improvements in cancer patient care and outcomes.
